# Detection of enterovirus 68 in serum from pediatric patients with pneumonia and their clinical outcomes

**DOI:** 10.1111/irv.12206

**Published:** 2013-11-10

**Authors:** Tadatsugu Imamura, Akira Suzuki, Socorro Lupisan, Taro Kamigaki, Michiko Okamoto, Chandra Nath Roy, Remigio Olveda, Hitoshi Oshitani

**Affiliations:** a Tohoku University School of MedicineSendai, Japan; b Research Institute for Tropical MedicineMuntinlupa City, Philippines

**Keywords:** Enterovirus 68, pediatric pneumonia, viremia

## Abstract

Enterovirus 68 (EV68) infection occasionally manifests with fatal outcomes. However, detection of EV68 in serum and its clinical outcomes are yet to be determined. In this study, we retrospectively tested stored serum samples collected from pediatric pneumonia patients whose nasopharyngeal specimens were positive for EV68. Of total 28 nasopharyngeal sample-positive patients, EV68 was detected in serum samples among 12 (43%) patients aged between 1 and 4 years. Our results suggest that EV68 can cause viremia by which the virus may exhibit systemic manifestations.

## Backgrounds

Human enterovirus 68 (EV68) was first isolated in 1962 from 4 pediatric patients hospitalized with lower respiratory infection, in California.^1^ Although this virus is a member of genus *Enterovirus*, in which most serotypes replicate and are shed in the gastrointestinal tract, EV68 is acid sensitive and grows more efficiently at the temperature of 33°C. Therefore, it is assumed that EV68 possess similar characteristics to human rhinoviruses (HRV) rather than enteroviruses.^2^ The detection of EV68 cases has increased substantially in recent years^3^ from different parts of the world, and in most cases, EV68 was associated with acute respiratory infections, including a considerable number of severe cases.^4^–^10^ A total of 5 fatal cases have been reported so far, including one pediatric patient who was admitted to emergency room with meningomyeloencephalitis in the United States.^11^ Systemic spread of EV68 from a respiratory tract was suggested as a possible pathogenesis for such severe infections.^11^ However, the mechanisms for systemic spread of EV68 are still unknown. Despite of increasing epidemiological importance of EV68, its pathogenesis is not yet completely understood.

Therefore, the objective of this study was to investigate whether EV68 could be detected in blood samples and the clinical course of the disease differed between patients, who were positive for EV68 in serum, and those who were negative.

## Study design

We retrospectively tested stored serum samples collected from patients whose nasopharyngeal specimens were positive for EV68. We conducted a prospective study for EV68 among pediatric patients in Leyte island, the Philippines, at Eastern Visayas Regional Medical Center (EVRMC) since May 2008. Nasopharyngeal swabs (EX-swab 002, DENKA SEIKEN, Tokyo, Japan) were collected from patients hospitalized at EVRMC with a diagnosis of severe acute respiratory infection (SARI), as defined by the World Health Organization.^12^ We previously detected EV68 in respiratory samples collected from 21 pediatric patients between October 2008 and February 2009,^4^ and from 9 pediatric patients between June and August 2011, using reverse transcriptase polymerase chain reaction (RT-PCR) targeting 5′ untranslated region (5′UTR) and viral protein 1 (VP1) region of EV68. In the study, serum samples were also collected at the same time along with nasopharyngeal samples from the pediatric patients who were hospitalized at EVRMC between May 2008 and December 2011. To avoid cross-contamination, the nasopharyngeal and serum samples were handled separately. The stored serum samples were tested for EV68 using RT-PCR targeting for 5′UTR and VP1 regions as described previously.^4^ Primer sets used for EV68 detection in respiratory and serum samples are listed in Table S1.

Patient information including demographics, symptoms, radiographic findings, O2 saturation level (SpO2), and outcomes was collected. Data were analyzed using jmp pro 9.0.2 software (SAS Institute Inc., Cary, NC, USA).

## Results and Discussion

Of total 30 pediatric patients, who were positive for EV68 in respiratory samples, serum samples were available for 28 patients (Table S2). Among these 28 serum samples, 5′UTR of EV68 was detected in 12 samples (Table S2), which represented 42·9% (12/28) of the collected serum samples. The 5′UTR sequences detected in serum samples were completely identical to those detected in respiratory samples of the same patient. The 5′UTR sequences detected from each patient had high similarity to each other, which was in line with previous studies from France^13^ and the United States.^5^ In this study, patients positive for 5′UTR of EV68 in serum samples were denoted as serum positive.

On the contrary, the VP1 of EV68 was amplified only in 5 serum samples. Notably, VP1 sequences were not also detected in some of the respiratory samples positive for 5′UTR of EV68, which was in line with our previous report,^4^ possibly due to the low virus titer in the clinical samples, or the highly varied sequences in VP1 region as it is the most variable genome region of enterovirsues.^14^ The VP1 sequences detected in serum samples were completely identical to those detected in respiratory samples of the same patient, and the detected VP1 sequences had unique nucleotide mutations, which differentiated them from each other completely.

**Figure 1:**
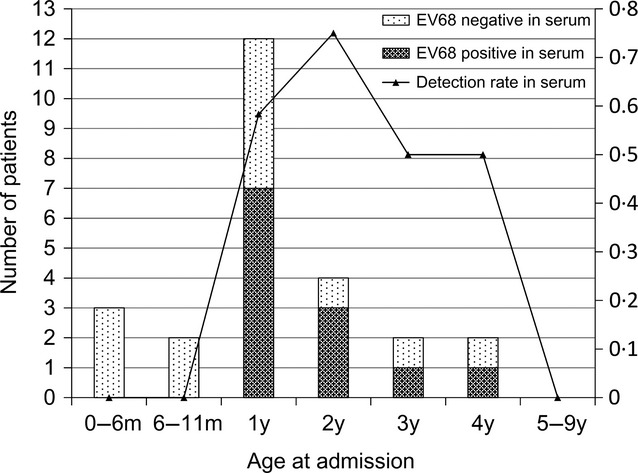
EV68 detection in serum and age distribution of pediatric patients who were positive for EV68 in serum, and those who were negative. *m; months, y; years.

The EV68 serum-positive patients were aged between 1 and 4 years, and remarkably, EV68 was not detected among those who were younger than 1 year of age (Figure 1).

We compared the patient's characteristics, including demographics (age younger than 1 year and sex), symptoms (presence of cough, chest indrawing, difficulty in breathing, wheezing, inability to drink or feed, cyanosis, irritability, drowsy, and coma), radiographic findings (presence of infiltration and consolidation), O2 saturation level, clinical outcome (fatal vs. discharged), and duration between onset of symptoms and hospital admission in between the patients who were EV68 positive (*n* = 12) and negative (*n* = 16) in serum. There was no significant difference in sex distribution, symptoms, radiographic findings, O2 saturation level, and clinical outcome (Table 1). The age younger than 1 year old was negatively associated with EV68 positivity in serum (*P* = 0·053). There was also no significant difference between the clinical presentations of EV68 serum-positive and EV68 serum-negative patients. In chest X-ray findings, infiltration and consolidation were more commonly observed among the EV68 serum-positive patients; however, the number of serum-positive patients was only two.

**Table 1:** Clinical manifestations of 28 pediatric patients whose serum samples were tested for EV68*

	EV68 positive in serum (*n* = 12)	EV68 negative in serum (*n* = 16)	*P*-value**
Demographics			
Younger than 1 year of age	0	5	0·0525
Sex (number of male)	5 (41·7%)	10 (62·5%)	0·4454
Symptoms			
Cough	12 (100%)	16 (100%)	NC
Chest in drawing	12 (100%)	16 (100%)	NC
Difficulty in breathing	11 (91·7%)	12 (75·0%)	0·3553
Wheezing	6 (50·0%)	11 (68·8%)	0·4410
Inability to drink or feed	4 (33·3%)	4 (25·0%)	0·6908
Cyanosis	1 (8·3%)	0 (0%)	0·4286
Irritability	2 (16·7%)	1 (6·3%)	0·5604
Drowsy	1 (8·3%)	2 (12·5%)	1·0000
Coma	1 (8·3%)	0 (0%)	0·4286
Chest X-ray findings			
Chest X-ray radiograph available	11 patients/12	14 patients/16	NC
Infiltration	3 (25·0%)	3 (21·4%)	1·0000
Infiltration and consolidation	2 (18·2%)	0 (0%)	0·1833
O2 saturation level at admission			
Before O2 treatment or bronchodilator	8 patients/12	10 patients/16	1·0000
Range (median)	88–100% (91·5%)	86–97% (92%)	>0·05
Outcome			
Died	1 (8·33%)	1 (6·25%)	1·0000
Duration between onset and consultation			
Range (median)	1–7 days (2 days)	2–14 days (4 days)	<0·05

* NC, not calculated.

** Statistical significance was calculated by Fisher's exact test or Wilcoxon rank sum test.

**Figure 2:**
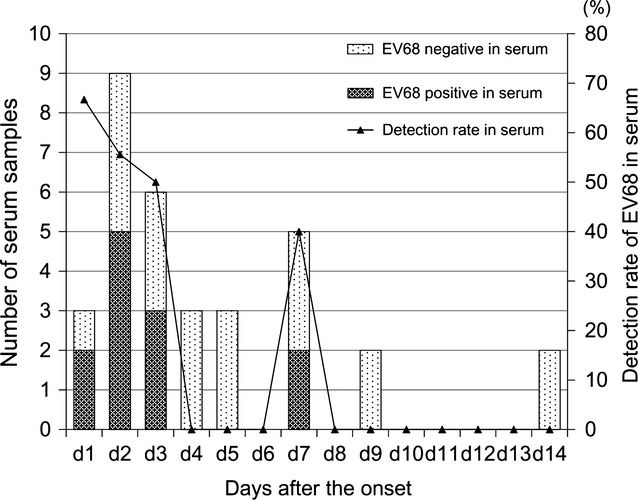
EV68 detection in serum and days between onset and hospital admission. *Positive rate of EV68 in serum on the day after the onset of symptoms are indicated on the top of each bar.

The EV68 serum-positive patients were admitted to EVRMC at 1-7 days (median, 2 days) after the onset of symptoms, while serum-negative patients were admitted at 2–14 days (median, 4 days) after the onset. The mean duration between onset of symptoms and hospital admission was significantly shorter (*P* < 0·05) among EV68 serum-positive patients compared with serum-negative patients. The positive rate of EV68 in serum was highest (66·7%, 2/3) among the patients who visited EVRMC on the next day of onset, and it decreased as the duration between onset and consultation became longer (Figure 2).

We did not find any significant difference in any of the clinical presentations between EV68-positive and EV68-negative pediatric patients, except for the duration between onset of symptoms and hospital admission.

Given a condition of two categories of younger than 1 year and older than 1 year, patients younger than 1 year were negatively associated with EV68 serum positives (*P* = 0·053). It could be possible that there might be some protective factors for pediatric patients younger than 1 year of age, such as the presence of maternal antibodies. However, considering the limited number of EV68-positive patients, and lack of EV68 detection in serum collected from patients aged between 6 months and 1 year, this could not be conclusive.

It is noteworthy to mention that we tested serum samples collected at the same hospital from two adult pneumonia patients, who were EV68 positive in respiratory samples (Table S1). However, both patients were found to be EV68 serum negative (data not shown). As the number of adult patients was too small to make any conclusions, further investigation should be carried out to reveal the association between patient age and EV68 positivity in serum.

Detection of EV68 in serum suggests that EV68 may cause viremia. However, we only detected partial genome of EV68 by PCR, and virus isolation from serum is required to confirm the existence of viremia. We previously reported that detection rate of human rhinovirus C (HRV-C) in serum was significantly higher than that of HRV-A and HRV-B, suggesting that HRV-C might have different pathogenicity compared with HRV-A and HRV-B.^15^ It was previously reported that EV68 has similar characteristics to HRVs,^2^ and notably, EV68 had similar frequency of virus detection in serum compared with HRV-C in our study. However, HRV-C-associated viremia has been poorly documented^16^; therefore, it is still elusive if there are any similarities in pathogenesis between EV68 and HRV-C. Moreover, the specificity of the EV68 serum-positive samples may also need to be further validated.

In conclusion, we reported the detection of EV68 in serum. Our results suggested that EV68 might be capable of entering into the blood, by which they might cause systemic spread. However, considering the fact that we detected EV68 using molecular methods, further studies are required to confirm the existence of viremia and systemic infection and to reveal the clinical significance of them.

## Supporting Information

**Table S1.** Primers used for detection and analysis of Enterovirus 68.

**Table S2.** Number of patients whose serum samples were tested and were positive for 5′UTR of EV68.

## References

[1] Schieble JH, Fox VL, Lennette EH (1967). A probable new human picornavirus associated with respiratory diseases. Am J Epidemiol.

[2] Oberste MS, Maher K, Schnurr D (2004). Enterovirus 68 is associated with respiratory illness and shares biological features with both the enteroviruses and the rhinoviruses. J Gen Virol.

[3] Centers for Disease C, Prevention (2011). Clusters of acute respiratory illness associated with human enterovirus 68–Asia, Europe, and United States, 2008-2010. MMWR.

[4] Imamura T, Fuji N, Suzuki A (2011). Enterovirus 68 among children with severe acute respiratory infection, the Philippines. Emerg Infect Dis.

[5] Tokarz R, Firth C, Madhi SA (2012). Worldwide emergence of multiple clades of enterovirus 68. J Gen Virol.

[6] Meijer A, van der Sanden S, Snijders BE (2012). Emergence and epidemic occurrence of enterovirus 68 respiratory infections in The Netherlands in 2010. J Virol.

[7] Piralla A, Baldanti F, Gerna G (2011). Phylogenetic patterns of human respiratory picornavirus species, including the newly identified group C rhinoviruses, during a 1-year surveillance of a hospitalized patient population in Italy. J Clin Microbiol.

[8] Ikeda T, Mizuta K, Abiko C (2012). Acute respiratory infections due to enterovirus 68 in Yamagata, Japan between 2005 and 2010. Microbiol Immunol.

[9] Linsuwanon P, Puenpa J, Suwannakarn K (2012). Molecular epidemiology and evolution of human enterovirus serotype 68 in Thailand, 2006-2011. PLoS One.

[10] Lauinger IL, Bible JM, Halligan EP, Aarons EJ, MacMahon E, Tong CY (2012). Lineages, sub-lineages and variants of enterovirus 68 in recent outbreaks. PLoS One.

[11] Kreuter JD, Barnes A, McCarthy JE (2011). A fatal central nervous system enterovirus 68 infection. Arch Pathol Lab Med.

[12] Ortiz JR, Sotomayor V, Uez OC (2009). Strategy to enhance influenza surveillance worldwide. Emerg Infect Dis.

[13] Renois F, Bouin A, Andreoletti L (2013). Enterovirus 68 in pediatric patients hospitalized for acute airway diseases. J Clin Microbiol.

[14] Oberste MS, Maher K, Kilpatrick DR, Pallansch MA (1999). Molecular evolution of the human enteroviruses: correlation of serotype with VP1 sequence and application to picornavirus classification. J Virol.

[15] Fuji N, Suzuki A, Lupisan S (2011). Detection of human rhinovirus C viral genome in blood among children with severe respiratory infections in the Philippines. PLoS One.

[16] Miller EK, Mackay IM (2013). From sneeze to wheeze: what we know about rhinovirus Cs. J Clin Virol.

