# Supplementary material for: Detection of enterovirus 68 in serum from pediatric patients with pneumonia and their clinical outcomes
Source: Influenza Other Respir Viruses. 2013 Nov 10;8(1):21–4. doi: 10.1111/irv.12206 (PMC4177794; doi:10.1111/irv.12206)
Supplement: Supplementary file 1 — Table S1. Primers used for detection and analysis of Enterovirus 68. [file irv0008-0021-SD1.docx]

Supplementary Table 1. Primers used for detection and analysis of Enterovirus 68

| Primer’s name (ref) | Primer’s sequence (5’ → 3’) | Location (Location number*) |
| --- | --- | --- |
| DK001 | CAAGCACTTCTGTTTCCC | 5’UTR (164-168) |
| DK004 | CACGGACACCCAAAGTAGT | 5’UTR (483-501) |
| 484 | GGRTCYCAYTACAGGATGT | VP1 (2197-2215) |
| 222 | CICCIGGIGGIAYRWACAT | VP1 (2933-2951) |
| EV68-VP1F | ACCATTTACATGCAGCAGAGG | VP1 (2393-2413) |
| EV68-VP1R | GACAAGAACTTTTTCAAATGGACAA | VP1 (2683-2707) |
| 485 | ACATCTGAYTGCCARTCYAC | 2A (3425-3406) |

* Location number is corresponding to the genome of EV68 Fermon strain (AY426531)
