# Supplementary material for: Detection of enterovirus 68 in serum from pediatric patients with pneumonia and their clinical outcomes
Source: Influenza Other Respir Viruses. 2013 Nov 10;8(1):21–4. doi: 10.1111/irv.12206 (PMC4177794; doi:10.1111/irv.12206)
Supplement: Supplementary file 2 — Table S2. Number of patients whose serum samples were tested and were positive for 5′UTR of EV68. [file irv0008-0021-SD2.docx]

Supplementary Table 2. Number of patients whose serum samples were tested and were positive for 5’UTR of EV68

|  |  | EV68 positive in  respiratory samples | Serum samples tested | Serum positive  for EV68 | % positive for EV68 in serum |
| --- | --- | --- | --- | --- | --- |
| 2008-09 | Pediatric | 21 | 19 | 9 | 47.4 |
|  | Adult | 0 | 0 | 0 | 0 |
| 2011 | Pediatric | 9 | 9 | 3 | 33.3 |
|  | Adult | 2 | 2 | 0 | 0 |
| Total | Pediatric | 30 | 28 | 12 | 42.9 |
|  | Adult | 2 | 2 | 0 | 0 |

Serum samples were collected from 30 including 28 pediatric patients (2008; 18 samples, 2009;1, and 2011;9) and 2 adult patients (2011), among which 5’UTR of EV68 was detected in serum collected from 12 pediatric patients (2008; 8 patients, 2009;1, and 2011;3).
